# Optimising the paradigms of human AI collaborative clinical coding

**DOI:** 10.1038/s41746-024-01363-7

**Published:** 2024-12-19

**Authors:** Yue Gao, Yuepeng Chen, Minghao Wang, Jinge Wu, Yunsoo Kim, Kaiyin Zhou, Miao Li, Xien Liu, Xiangling Fu, Ji Wu, Honghan Wu

**Affiliations:** 1 School of Computer Science (National Pilot Software Engineering School), Beijing University of Posts and Telecommunications, Beijing, China; 2 Key Laboratory of Trustworthy Distributed Computing and Service (BUPT), Ministry of Education, Beijing, China; 3 Institute of Health Informatics, University College London, London, UK; 4 Department of Electronic Engineering, Tsinghua University, Beijing, China; 5 College of AI, Tsinghua University, Beijing, China; 6 School of Health and Wellbeing, the University of Glasgow, Glasgow, UK

**Keywords:** Health services, Information technology

## Abstract

Automated clinical coding (ACC) has emerged as a promising alternative to manual coding. This study proposes a novel human-in-the-loop (HITL) framework, CliniCoCo. Using deep learning capacities, CliniCoCo focuses on how such ACC systems and human coders can work effectively and efficiently together in real-world settings. Specifically, it implements a series of collaborative strategies at annotation, training and user interaction stages. Extensive experiments are conducted using real-world EMR datasets from Chinese hospitals. With automatically optimised annotation workloads, the model can achieve F1 scores around 0.80–0.84. For an EMR with 30% mistaken codes, CliniCoCo can suggest halving the annotations from 3000 admissions with an ignorable 0.01 F1 decrease. In human evaluations, compared to manual coding, CliniCoCo reduces coding time by 40% on average and significantly improves the correction rates on EMR mistakes (e.g., three times better on missing codes). Senior professional coders’ performances can be boosted to more than 0.93 F1 score from 0.72.

## Introduction

Clinical coding, as the fundamental task of transforming medical information written in electronic medical records (EMRs) by clinicians into structured codes, is a significant component of clinical research and billing management^1^. Traditional clinical coding task is a resource-intensive process which requires a group of specialised clinical coders to manually conduct systematic code assignments for multi-source, multi-modal raw medical records based on standard coding classification systems consisting of thousands of candidate codes^2^. For example, the most predominant coding classification systems is the ICD-10 (International Classification of Diseases, Tenth Revision) which contains around 68,000 diagnosis codes^3^. As a result, the whole coding process is expensive, time-consuming, and error-prone. According to corresponding statistics^4^ and our interview with clinical coders in Tsinghua Changgung Hospital, a clinical coder in a Chinese hospital usually codes about 300 cases a month, i.e., 32–34 min per case as a full-time coder. Such labour-intensive coding tasks may often accumulate to a backlog of uncoded cases for months to clear^5^. In addition, the sampling survey conducted by the National Health Commission of the PRC (NHC China) in 2020^6^ reveals the average accuracy of diagnosis coding in tertiary and secondary hospitals is only about 66.6%, which is clearly not ideal for enabling further healthcare administration and improvements as well as secondary use for research^7^.

Giving the inefficiency and ineffectiveness of manual clinical coding, automated clinical coding (ACC) has been considered a promising approach to facilitate the management of medical records and clinical research using routinely collected health data. In the natural language processing (NLP) community, automated clinical coding is often treated as a text classification task, which can be simply formulated as *X* → *y*, where *X* is a piece of medical text from EMRs, and *y* ∈ [0, 1]^*n*^ is the label vector of the corresponding codes. ACC based on unstructured free text, e.g., discharge summary, can be defined as a multi-label text classification (MLTC) task, while ACC based on structured text, e.g., disease name and surgery name, can be defined as a multi-class text classification task. In recent years, a series of machine learning and deep learning-based methods for ACC, especially for automatic ICD coding, have been proposed^4,8–12^. These works focus on improving representation performance by designing different representation architectures to model the hierarchical knowledge^13–15^ and external medical knowledge^16,17^ of ICD taxonomy. Despite substantial developments and improvements in recent years, the performance of existing representation methods remains unsatisfactory for realising automated coding in real-world scenarios. For example, the micro-F1 score of MSMN^18^, one of the SOTA ACC approaches in 2023, on the MIMIC-III 50 benchmark^19^ is only 72.5%. As a result, the adoption of end-to-end AI-based coding systems in real-world settings is still rare, if exists at all.

Recently, the emergence of human-in-the-loop (HITL) learning paradigm has drawn wide and increasing attention from the medical informatics community^20–25^, which is viewed to as an exciting approach to unlocking the full potential of AI by combining human and machine intelligence^26,27^. Technically, HITL learning framework focuses on complex and risky decision-making tasks, aiming at a harmonious balance between efficiency and quality by incorporating human expertise with machine intelligence^28^, which aligns well with the principle of clinical decision support setting. Hence, HITL has been utilised for multi-modal clinical decision-making tasks, e.g., MRI-based knee lesion diagnosis, dialogue-based sentiment analysis, and ECG reading study^29,30^. HITL has also been embedded in the designs of clinical interfaces, providing task-specific, visualised, and customised interactions to optimise the explainability and usability of AI-based systems (section 2 in Supplementary Information)^20–22^. For practical deployments of ACC systems, Dong and colleagues suggested that it is essential to consider the HITL approach to involve coders’ feedback^1^. While there seems a consensus on HITL being a potential enabler, what is largely understudied is the question of how human and AI could work efficiently and effectively together on the challenge coding task.

In this paper, we propose a novel HITL framework, CliniCoCo, for human–AI Collaborative Clinical Coding in real-world scenarios. The proposed CliniCoCo involves clinical coders’ feedback in the key stages of the ACC system, i.e., data preprocessing stage, model training stage, and clinical decision-making stage, and fully considers the complex medical record characteristics and clinical process in Chinese hospitals. To our best knowledge, this is the first work that systematically designs a HITL paradigm for the task of ACC. The main contributions of this paper are summarised as follows:

- To minimise the workload and maximise the effectiveness of manual annotations, we propose a HITL collaborative strategy for code annotation to implement an active-learning strategy. It involves the adoption of semi-automatic dynamic & iterative collaborative annotation module which effectively constructs automatic large-scale noisy-labelled dataset and adaptively constructs manual small-scale clean-labelled dataset.
- We propose a 3-step multi-label-oriented contrastive learning strategy to fully leverage labelled datasets with different noise levels so as to deeply enhance the representation performance of ACC. Moreover, with the feature extractor obtaining the capability of distinguishing the similarity between medical samples, the kNN-optimised inference module is designed to further involve coders’ priori medical knowledge from representative samples and optimise the prediction results in a HITL way.
- We design multiple customised collaborative functions in the clinical decision-making stage, including threshold adjustment, heatmap visualisation, and similar reference retrieval to consistently optimise the performance and usability of ACC in clinical scenarios. A HITL iterative interface is developed for CliniCoCo, which integrates and visualises the collaboration designs in the whole process.
- Extensive experiments conducted on the real-world EMR datasets we constructed from two tertiary Chinese hospitals demonstrate the effectiveness of CliniCoCo under diverse clinical settings. Extended quantitative analysis, pilot experiments, and interviews based on the HITL interface are conducted to test the utility and to observe the deployment of CliniCoCo in complex clinical scenarios.

## Preliminary background and overall framework

**Fig. 1:**
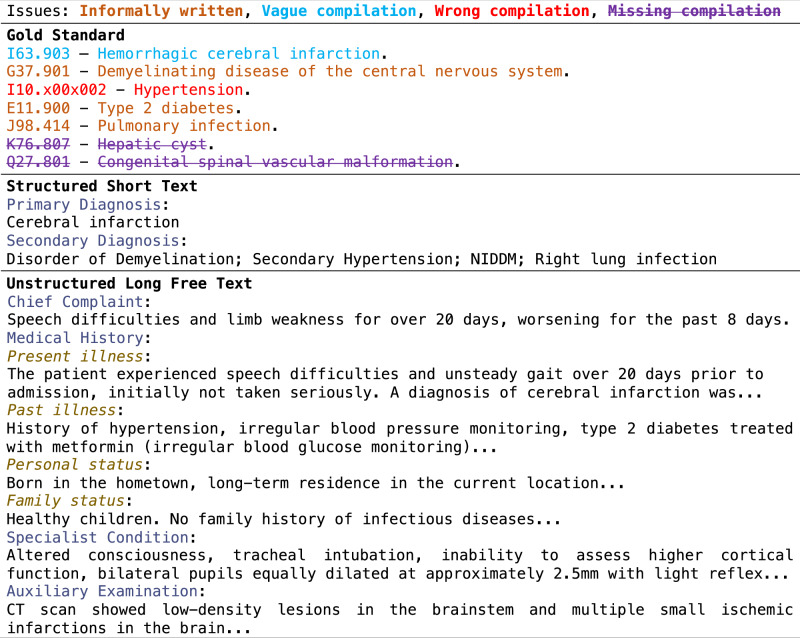
A translated semi-structured raw EMR sample from an Anhui provincial hospital, including gold standard for diagnosis codes and names, and main contributory records comprising structured short text and unstructured long free text. The colour coding and text styles in the gold standard indicate the types of corrections on the issues of informally written (using non-standardised terms) and three types of mistakes from the structured short text are marked.

The clinical coding process in Chinese hospitals involves a two-step process: in their routine care clinicians first fill free-text information into the EMR system; then, at the coding stage clinical coders assign ICD codes for each episode from the EMR data. For the coding task, the contributory information from summary pages of inpatient EMRs is twofold. One comprises an unstructured, long free text, describing chief complaint, medical history, specialist condition, and auxiliary examination^31^. The other comprises a structured short text, i.e., a list of names, including primary disease names, secondary disease names, and surgical procedures (section 1 in Supplementary Information). While the structured short text seems directly giving the coding information, however, it is often written informally (not following any controlled vocabularies), leading to the presence of a wide range of name variants for the same diseases or procedures. It may also contain inaccurate information, e.g., using a concept that is too broad (e.g., stroke without specifying either ischaemic or haemorrhagic), wrong disease/procedure names, or missing key diagnostic information. Consequently, structured short text alone cannot serve as the ground truth for coding. In this study, clinical codes derived from such data is considered as noisy labels. Figure 1 illustrates an anonymised and translated EMR example from an Anhui provincial hospital.

**Fig. 2:**
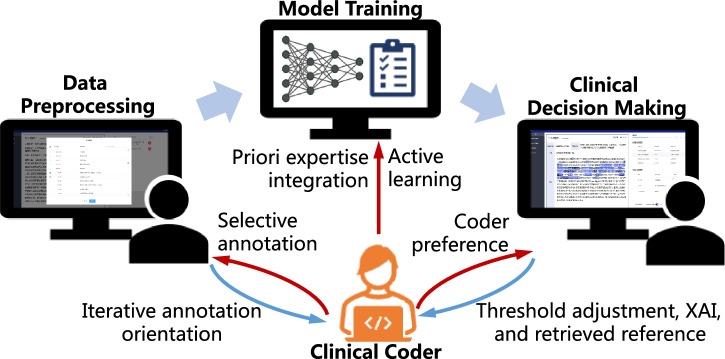
The overall HITL framework of CliniCoCo. Clinical coders and the AI-based coding system can deeply and mutually collaborate in all key stages of the coding process.

This paper proposes a HITL framework, CliniCoCo, for realising and optimising human–AI collaborative clinical coding for the aforementioned clinical coding procedure and EMR characteristics. The overall framework is depicted in Fig. 2. CliniCoCo implements a series of collaborative strategies to involve clinical coders’ inputs and feedback into all three stages of the automated coding process. At the data preprocessing stage, a dynamic and interactive process is implemented to seek inputs from human aiming for collecting sufficient human-labelled data with minimised human annotation efforts. At the training stage, a prior knowledge repository is derived from human annotations as pairs of vector representation and labels, which will facilitate improving the classification via a knowledge-driven approach. At the deployment stage, explainable AI and threshold adjustment mechanisms will assist clinical coders in coding with AI model’s attentions and customisable suggestions.

**Fig. 3:**
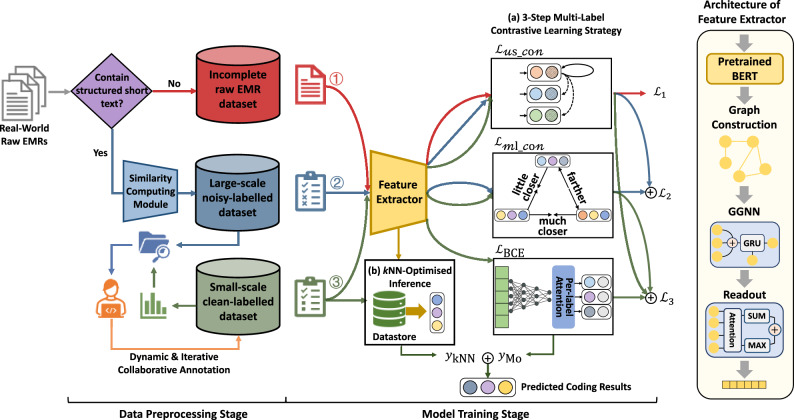
Architecture design and collaborative strategies of CliniCoCo. The framework comprises three stages: data preprocessing, model training and prediction, and clinical decision-making, which are illustrated in each sub-figure, respectively. (1) The data preprocessing stage gets inputs of raw EMRs and generates three types of datasets: incomplete raw EMR, noisy-labelled data and human-labelled data. (2) Using the three types of data outputs from stage 1, model training and prediction is composed of a (a) 3-step multi-label contrastive learning strategy and (b) a kNN-based inference optimisation module. (3) The model-enabled decision-making is conducted via an interactive interface supporting confidence-threshold adjustment, interpretation (heatmap) visualisation, and similar reference retrieval are integrated.

The overall architecture design and collaborative strategies of the CliniCoCo framework are shown in Fig. 3, which are described with details and formalisation in “Methods”. In summary, (1) in the data preprocessing stage, the dynamic and iterative collaborative annotation strategy is designed, where large-scale noisy-labelled dataset and small-scale clean-labelled dataset are automatically and adaptively constructed in a two-step workflow, respectively; (2) in the model training stage, the three-step multi-label contrastive learning training strategy is proposed to fully leverage medical knowledge from EMR datasets with the different noise level, and the kNN-based inference optimisation module is proposed to further involve coders’ priori expertise in the model prediction; (3) in the clinical decision-making stage, the interactive interface of CliniCoCo is developed, where a series of customise HITL functions are integrated, including threshold adjustment, heatmap visualisation, and similar reference retrieval.

## Results

CliniCoCo was trained and evaluated on real-world semi-structured EMRs from two Chinese tertiary hospitals. We first describe the curated datasets and detailed construction method. Then, we conduct comparative experiments and ablation studies to demonstrate the clinical utility of CliniCoCo and the effectiveness of the specific collaborative strategies designed in different modules. Furthermore, quantitative analysis and qualitative interviews will be presented on data collected from (a) pilot experiments in a real-world setting and (b) interviews with clinical coders.

### Datasets

**Fig. 4:**
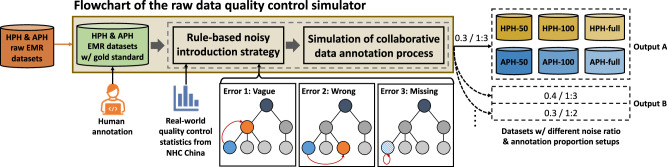
The flowchart of our proposed raw data quality control simulator utilised to construct the datasets in the experiments. Specifically, we first design a rule-based automatic raw dataset construction strategy to control the noise ratio and error types in the raw datasets and then simulate the collaborative data annotation process.

Two sets of datasets were used in our comprehensive evaluations, shown as output A and B on the right-hand side of Fig. 4. Briefly, *Output A* was used for evaluating the overall performance of automated coding models and *Output B* was for assessing different levels of noisy data and human annotations in affecting model performances. As depicted Fig. 4, these datasets were generated by *an EMR simulator*. The input to the simulator was real-world EMRs from two Chinese hoipitals: a Hebei provincial hospital (HPH) and an Anhui provincial hospital (APH). In the simulation process, the raw EMRs were first fully annotated by human experts to form the gold standard data. Such data then went through a two-step process to introduce different levels of noise and annotations for mimic different human–AI collaborative scenarios. The noise included three types of errors or mismatches: as shown at the bottom right of the figure, (a) vague—the correct diagnosis was replaced by a more general diagnostic code; (b) wrong—the correct diagnose was replaced by another not clinically relevant code; (c) missing—a diagnosis was removed. For *Output A*, we introduced a 30% noise and 1:3 annotated data (25% of the data was manually annotated), which was based on the NHC China’s 2020 report on the coding quality of EMRs across China^6^. This was to reflect the realistic situation of model performances in Chinese hospitals. Detailed statistics of the six datasets are listed in Table 1. The 50/100-code datasets are constructed based on the label distribution of the full set of ICD codes. We screened out primary diagnosis codes and other frequently distributed secondary diagnosis codes, while masked other diagnosis codes to make sure experimental conditions satisfy the HITL setups in the data preprocessing stage. For *Output B*, we generated a series of datasets using different levels of noise and annotations for assessing the performances changes of our human–AI collaborative framework, hopefully among others revealing the optimal collaborative setup. Details of the setups are described in the subsection “Quantitative Analysis of the Effort vs Effectiveness of Human Inputs”.

**Table 1:** Statistics of our constructed datasets from real-world EMRs in two Chinese hospitals

	HPH-50	APH-50	HPH-100	APH-100	HPH-full	APH-full
Number of						
# Doc.	10,223	9514	10,682	14,104	10,682	14,104
Avg # words per Doc.	728	1472	725	1051	725	1051
Avg # codes per Doc.	4.27	4.03	4.89	4.78	5.97	5.58
Total # codes	50	50	100	100	671	579
Completeness ratio of						
# Specialist condition	65.27%	60.15%	62.27%	60.51%	62.27%	60.51%
# Auxiliary examination	47.76%	83.67%	47.13%	63.04%	47.13%	63.04%

### Effectiveness of CliniCoCo in automated clinical coding

#### The effect of overall representation method

Specifically, we reproduced multiple representation architectures of the representative ACC baselines and utilised them to replace our proposed feature extractor in CliniCoCo, including CAML^16^, LAAT, Joint LAAT^13^, and BERT-based method proposed by Chen et al.^32^ (section 3 in Supplementary Information). The results are shown in Table 2. First, the overall HITL framework is applicable and effective for variants with diverse feature extractors to have consistent performance. Compared with all the variants in terms of F1 score, AUC, and Recall@5, our proposed CliniCoCo achieves best performance under all the HPH and APH dataset settings, which demonstrates the effectiveness of our designed feature extractor. Moreover, the improvement of CliniCoCo compared with the BERT-based approach (Chen et al.) demonstrates the contribution of the GNN and Attention-based architecture. The two combined enhance the representation by modelling the interactions of knowledge on contextualised representation from BERT. The overall performance achieved by CliniCoCo (AUC ranging 0.93–0.97, F1 0.80–0.84) indicates the whole architecture’s high effectiveness in facilitating the clinical coding task.

**Table 2:** Performance (%) in AUC, F1 score, and Recall@5 of CliniCoCo and the variants with different ACC baselines adopted as feature extractor and different noise-level datasets introduced for training

	HPH-50					APH-50				
	AUC		F1		Recall	AUC		F1		Recall
Variants	Macro	Micro	Macro	Micro	R@5	Macro	Micro	Macro	Micro	R@5
CAML	86.83	90.42	67.92	75.48	76.94	85.60	89.27	66.72	74.83	72.89
LAAT	91.35	93.61	77.23	81.19	80.64	90.27	92.82	76.16	80.22	78.64
Joint LAAT	91.35	93.61	76.78	81.85	81.70	90.27	92.82	76.01	81.05	79.35
Chen et al.	90.53	93.14	76.61	82.94	82.03	89.69	92.35	75.83	82.70	80.57
w/o noisy-labelled	84.72	88.42	62.69	70.79	71.38	83.88	87.09	63.42	70.36	67.88
w/o raw incomplete	92.11	94.26	80.45	83.03	82.83	90.84	93.10	78.99	82.45	80.29
CliniCoCo	92.16	94.29	80.82	84.20	84.13	91.07	93.32	79.64	83.67	81.48

	HPH-100					APH-100				
	AUC		F1		Recall	AUC		F1		Recall
Variants	Macro	Micro	Macro	Micro	R@5	Macro	Micro	Macro	Micro	R@5
CAML	88.56	92.04	64.20	75.26	74.82	90.31	94.87	64.05	74.92	75.23
LAAT	93.02	95.23	72.31	79.84	78.90	93.52	96.26	71.28	78.39	78.46
Joint LAAT	93.02	95.23	72.28	80.25	79.27	93.52	96.26	71.24	78.83	78.80
Chen et al.	92.97	95.65	71.85	80.97	79.38	91.69	95.33	70.83	79.24	79.32
w/o noisy-labelled	85.73	88.13	61.30	68.75	67.94	84.83	89.30	60.57	66.97	67.03
w/o raw incomplete	93.20	95.51	74.96	81.06	79.30	94.86	96.98	73.09	79.51	79.64
CliniCoCo	93.34	95.73	75.49	82.25	80.01	95.02	97.16	73.72	80.43	80.47

The results of CliniCoCo in full-size scenarios (i.e., including all ICD-10 codes for training and test) are shown in Table 3. With a large amount of uncommon diagnosis codes involved, our proposed CliniCoCo still performs robustly and effectively with micro-F1 scores reaching 0.7336 and 0.7665. However, the macro F1 scores show a significant decrease on the full-size datasets compared to the 50/100-label datasets. This drop is due to the few-shot nature of many uncommon diagnosis codes in the full-size datasets and the limitation of the experimental conditions that the curated samples cannot fully support our proposed dynamic and iterative collaborative annotation strategy for all diagnosis codes. That is, there are none or few candidate cases in the dataset available for the collaborative annotation, meaning many codes were never seen by the model in training. In real-world scenarios, with a wider range of raw EMRs curated, the effectiveness of our proposed HITL strategies for data annotation would be further reflected for these few-shot codes. In addition, given that the Recall@9 on the two datasets both exceed 0.82, the limitation on few-shot codes can be further optimised based on the collaborative coding mechanisms designed in the clinical decision-making stage.

**Table 3:** Performance in AUC, F1 score, and Recall@7/9 of CliniCoCo on full-label datasets

		AUC		F1		Recall	
		Macro	Micro	Macro	Micro	R@7	R@9
CliniCoCo	HPH-full	0.9501	0.9796	0.2484	0.7665	0.7815	0.8222
	APH-full	0.9596	0.9842	0.1994	0.7336	0.7900	0.8276

#### The effect of introduced noisy datasets

As is shown in Table 2, the introduction of the noisy-labelled dataset and raw incomplete dataset both contribute to the performance improvements of CliniCoCo, which demonstrates that the rich medical knowledge implicit in the noisy datasets are effectively leveraged through our proposed 3-step multi-label contrastive learning strategy. Notably, the large-scale noisy-labelled dataset has a more significant effect on CliniCoCo than the raw incomplete dataset. Given the similar numbers of samples they contain, it can be reasoned that unsupervised contrastive learning has a weaker impact on the clinical free text, while our proposed MLTC-oriented supervised contrastive learning seems more capable at knowledge mining with the intervention of noisy labels.

#### The effect of collaborative annotation strategy

**Fig. 5:**
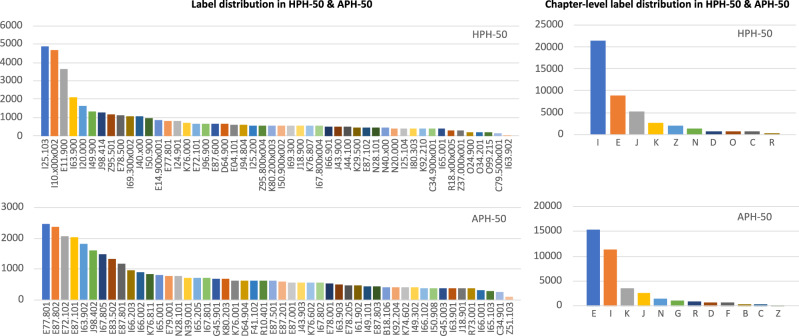
Statistics of fine and coarse-grained label distribution in the datasets HPH-50 and APH-50. The coarse-grained labels denote clinical codes in units of chapters. Long-tailed label distribution can be observed in both datasets.

Given the uneven distribution characteristics of clinical codes in dataset, from the third aspect, we further evaluate the effectiveness of our proposed HITL framework in units of chapter-level diagnoses by introducing collaborative annotation strategy in the data preprocessing stage. Figure 5 presents the fine and coarse-grained label distributions of HPH-50 and APH-50, while Table 4 presents corresponding chapter-level performance in the two datasets. Specifically, in the variant without collaborative annotation setting, we only keep the initialisation operation of patch selection, where 35 candidate samples of each label are selected from the noisy-labelled dataset for further annotation, while the left candidate samples are all randomly selected. It can be seen that the performance of CliniCoCo varies among different chapters of diseases, which is overall consistent with the long-tailed effect of the datasets revealed in Fig. 5. However, all main chapter-level performances are robust. This may benefit from our designed dynamic and iterative collaborative annotation strategy which adaptively orients the manual annotation based on the statistics of label distribution in a clean-labelled dataset. In particular, more substantial improvements are observed in chapters with small samples, especially those chapters in the second and third rows of the table.

**Table 4:** Chapter-level coding performance in micro-F1 score of CliniCoCo and its variant without adopting collaborative annotation strategy

Setting	Dataset	Chapter-level diagnosis						
		I	E	J	K	Z	N	D
CliniCoCo	HPH-50	0.8473	0.8689	0.7943	0.8244	0.8573	0.8254	0.8241
	APH-50	0.8238	0.8582	0.8252	0.7992	0.7211	0.7864	0.8672
Collaborative annotation w/o	HPH-50	0.8475	0.8693	0.8012	0.8210	0.8302	0.8067	0.8087
	APH-50	0.8218	0.8611	0.8179	0.7956	0.6883	0.7745	0.8423
Difference value	HPH-50	−0.0002	−0.0004	−0.0069	+0.0034	+0.0271	+0.0187	+0.0154
	APH-50	+0.0021	−0.0029	+0.0073	+0.0036	+0.0328	+0.0119	+0.0249
		O	C	R	G	B	F	
CliniCoCo	HPH-50	0.7188	0.8117	0.7824	N/A	N/A	N/A	
	APH-50	N/A	0.7455	0.7486	0.8287	0.8213	0.8286	
Collaborative annotation w/o	HPH-50	0.6925	0.7946	0.7506	N/A	N/A	N/A	
	APH-50	N/A	0.7222	0.7264	0.8043	0.8002	0.8019	
Difference value	HPH-50	+0.0263	+0.0171	+0.0318	N/A	N/A	N/A	
	APH-50	N/A	+0.0233	+0.0222	+0.0244	+0.0211	+0.0267	

Specific titles of each chapter are listed at the bottom of the table.

### Quantitative analysis of the effort vs effectiveness of human inputs

To analyse the trade-off between annotation efforts and their overall effectiveness in facilitating ACC, here we conduct experiments by quantifying the annotation quality from two perspectives of dataset characteristics, i.e., the proportion between datasets with different noise levels and the noise ratio in the noisy raw datasets.

**Fig. 6:**
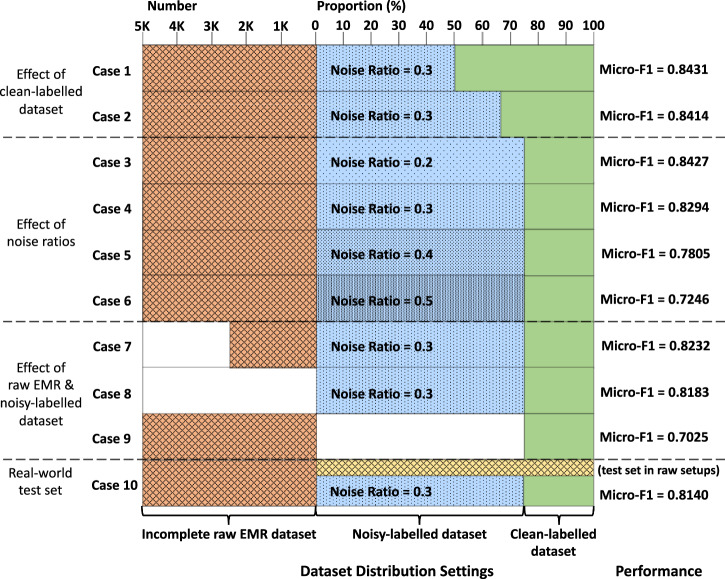
Detailed dataset distribution of ten cases and their corresponding micro-F1 performance on HPH-50 without kNN-based inference involved, where the number of the samples in incomplete raw EMR dataset, the proportion between noisy and clean-labelled dataset, and the noise ratio in the labelled dataset are adjusted, respectively. The number and proportion at the top denote the metrics for quantifying the size and distribution of the three datasets with different noise levels. The textures indicate the noise in the three types of datasets.

Specifically, we sampled ten cases of dataset annotation settings on HPH-50. The detailed dataset distribution, noise ratio, and corresponding F1 score of each case are shown in Fig. 6. To reflect the effect of dataset distribution on model training more explicitly, here we get rid of the kNN-based inference and only present the feature extractor-based performance. First, it can be seen from case 3 and 9 that the proportion of clean-labelled dataset, namely human-based annotation efforts, still plays a crucial role in the process of representation training. However, the results of case 1, 2 and 4 reveal that owing to the enhancement by contrastive learning training strategy, the continuous augmentation of the noisy-labelled dataset introduced in the training process does alleviate the drawback of limited scale of the clean-labelled dataset to a great extent, which exactly caters to our objective to release the lacked labour of manual annotation. Quantitatively, for HPH-50 EMR dataset with 30% noise ratio setting, the optimal annotation effort is labelling 1500 admissions, i.e., 1:3 manual versus automatic annotation proportion, which can achieve a F1 score of 0.8294, while more manual labelling than this, however, is not cost-effective. For example, 1500 more annotated admissions would only increase the F1 score by 0.0137. Meanwhile, based on case 4, 7 and 8, the introduction of raw incomplete dataset works only when the sample quantity reaches a certain magnitude, which is expected to take bigger effect in real-world scenarios with more accessible raw EMRs introduced.

Last but not least, an obvious negative correlation between the noise ratio in the raw datasets and the representation performance can be observed from cases 3 to 6, which reveals that the performance of CliniCoCo is sensitive to the quality of structured short text in raw EMRs. That is to say, the coding performance of the HITL framework directly depends on the normalisation and completeness of raw records written by clinicians. Despite recognising the significance to strengthen the writing skills and lower the noise ratio in raw EMR text, we need to further seek the trade-off between the efforts and effectiveness of clinicians’ inputs from the perspective of clinical EMR management. More specifically, as is shown in Fig. 6, a marked slowdown in growth of the performance with the decrease of noise ratio can be observed, which reveals the noise ratio ranging from 0.2 to 0.3 as a potentially most cost-effective benchmark for clinicians to keep balance between coding efficiency and coding quality regarding EMR management, and thereby can be treated as a quantified metric for further EMR quality improvement. Furthermore, in case 10, we used the model trained on a setup of case 4 and evaluated the performance on a test set extracted from the raw EMRs to evaluate model effectiveness in a real-world scenario. CliniCoCo achieves a F1 score of 0.8140, within the region of F1 scores between cases 4 and 5. This implies that the real-world EMRs having an error rate ranging from 0.3 to 0.4.

### Pilot experiments and interviews

Apart from evaluating the effectiveness of CliniCoCo from the perspective of representation performance, more attention should we pay to observe the clinical deployment and interaction of the HITL framework. Therefore, our aim is further extended to address the question: “How does CliniCoCo perform to realise human–AI collaboration when deployed in real-world clinical coding scenarios?”. We conducted a real-world pilot study involving nine clinical coders from an independent tertiary hospital in Jiangsu Province, China, to evaluate the effect of our proposed HITL framework in a real-world setting with diverse scenarios.

#### Experimental settings

**Fig. 7:**
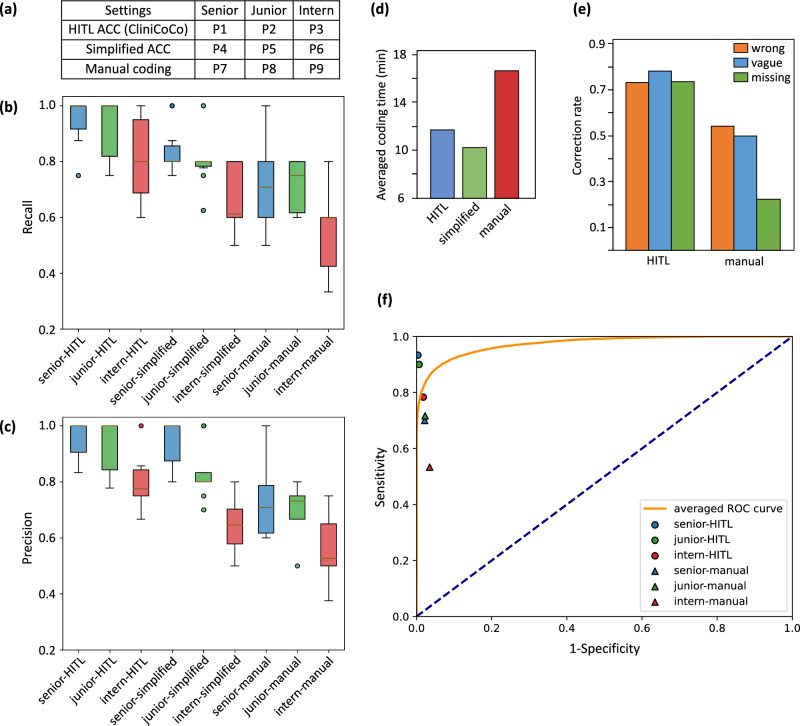
Fine-grained evaluations on the coding results from the pilot study. **a** Distribution of the experimental setups for the nine participants in the pilot study. Box plots for **b** the recall rates and **c** the precision rates of the nine participants. **d** Averaged coding time under three coding modes. **e** Averaged correction rates for three error types under the HITL ACC mode and the manual mode. **f** Averaged ROC curve of CliniCoCo and coding distribution of the participants under HITL ACC mode and manual mode.

For human participants, a total of nine coders are recruited. Based on their years of working experience, the participants were divided into 3 groups, i.e., seniors (more than 5 years), juniors (1–5 years), and interns (less than 1 year). Each participant was tasked to use the system in only one of the three configurations (detailed below). All participants worked on the same set of 10 EMR samples. Such 10 EMRs were randomly selected from the test set of HPH-100 following two criteria: (1) Each diagnosis code should appear up to two times in the set, and (2) the samples should contain codes with all high (top 15), middle (ranked No. 16–45), and low-frequencies (ranked No. 46–100) according to the label distribution in HPH-100. Before the experiment started, the participants had a chance try out the system and get familiar with different functions using an independent EMR sample. Detailed distribution of the experimental setups in the pilot study are shown in Fig. 7a.

Regarding the coding scenarios, three distinct configurations were designed, including (1) HITL ACC mode, (2) simplified ACC mode, and (3) manual coding mode. Specifically, the HITL ACC mode is exactly based on our proposed framework CliniCoCo to conduct human–AI collaborative clinical coding. The simplified ACC mode gets rid of all the collaborative strategies and customised functions designed in the key stages of ACC, thereby only providing “black-box” AI-based prediction results for coding support. The manual coding mode performs as a traditional hospital information system which presents only raw EMR dataset and requires coders to retrieve and select all related clinical codes.

#### Quantitative analysis

For evaluating the effectiveness among different configurations, Fig. 7b–d illustrates the results quantified by recall, precision, and time used for coding. Specifically, both ACC settings led to higher recall and precision rates among coders with varying levels of experience, while coders operating under the HITL ACC mode further outperformed those using the simplified mode, highlighting the effectiveness of collaborative strategies in key stages of the ACC process. In terms of efficiency, both ACC settings required shorter coding time. They took around 10–12 min per case, which was about 30–40% reduction of time needed per case compared manual coding. However, the averaged coding time under HITL ACC mode was slightly longer than that of the simplified ACC mode, which may be due to the adjustment operations of customised functions integrated in the interface.

We further evaluated the inter and intra-variability. Specifically, inter-variability depicts how clinical coders deal with noisy EMR data, while intra-variability depicts how clinical coders with different coding experience. From inter-variability, the impact of human–AI collaborative coding on different types of coding errors is illustrated in Fig. 7e. Compared with the HITL mode, the manual coding mode demonstrated significant (50% on average) lower accuracy on cases with errors, particularly for missing codes. From intra-variability, the most significant improvement was observed among intern coders with 0.26 increases in recall and 0.25 increases in precision on average (see Fig. 7b, c). However, as shown in Fig. 7f, the collaborative coding results performed by intern coders still fall short compared to the averaged ROC curve which denotes the performance only based on CliniCoCo framework. This discrepancy is primarily due to the interns’ limited coding knowledge. In contrast, the senior and junior clinical coders outperform both the human-only and AI-only setups, which demonstrates the positive impact of collaborative coding approach. Within each setup, the intern-HITL had the highest recall variability (0.25, Q3–Q1 on recall) and junior-simplified had the lowest (0.04). The intern-manual had the highest precision variability (0.26) and senior-HITL had the lowest (0.15). Overall, senior-HITL had the highest recall (0.95) and precision (0.95) with a small in-group variance.

#### Qualitative interview

Furthermore, we interviewed the clinical coders and interns to assess the usability of our developed interfaces for CliniCoCo in real-world scenarios. Detailed preferences and evaluations of the interviewees are summarised as below. (1) Regarding the layout of the system, they prefer separated presentation of the predicted results based on structured short text and long free text, which would make the predicted results easy to understand and compatible with traditional coding process. (2) In addition, they affirm the function which presents the retrieved representative similar samples from the datastore as reference, which may be contributive especially for the less experienced clinical coders. (3) Regarding the threshold adjustment function, they tend to prefer turning down the threshold a little bit for better recall performance. This may be because they want more information provided due to trust issue and to reduce search operations. (4) All interviewees give an overall positive assessment on CliniCoCo in terms of its clinical utility for human–AI collaboration. Given the unsatisfactory status of clinical coding in Chinese hospitals^6^, it is expected that the deployment of CliniCoCo would optimise the coding efficiency and quality by a large margin, especially in the secondary hospitals.

## Discussion

This study focuses on optimising the adoption and deployment of ACC systems in complex real-world clinical scenarios which involves numerous unexploited noisy raw clinical text and frequent coder-computer interaction for clinical decision-making, proposing a novel HITL framework CliniCoCo to transfer the paradigm from automated to human–AI collaborative clinical coding.

To incorporate clinical coders’ inputs effectively and efficiently, the three key stages of ACC systems are designed in CliniCoCo to fit with the medical record characteristics and facilitate clinical coding process in real-world scenarios. First, to minimise manual labelling effort, the dynamic and iterative collaborative annotation strategy is designed in data preprocessing stage to automatically construct large-scale noisy-labelled dataset and adaptively construct small-scale clean-labelled dataset, while the 3-step MLTC-oriented contrastive learning training strategy and the similar reference retrieval module are designed in model training stage to sequentially leverage the medical knowledge implicit in datasets with different noise levels and priori samples. Therefore, the annotation workload is minimised while the coding effectiveness is maximised. Second, to utilise human’s knowledge in improving the collaborative coding accuracy, a series of HITL functions are designed in the clinical decision-making stage and are integrated in an interactive interface. All these combined are aimed to optimise the performance, explainability, and usability of the coding system to fit in complex clinical coding decision support scenarios.

Different to studies pursuing the improvements on the SOTA performance of AI-based ACC methods, this study aims to establish a general paradigm for human–AI collaborative clinical coding that is compatible with and adaptable to diverse complex real-world clinical scenarios. Therefore, to comprehensively evaluate CliniCoCo under different clinical circumstances, an EMR data quality control simulator is designed to populate EMRs with different noise ratio and error types as well as different levels of annotations.

Based on the constructed datasets, experiments in “Results” evaluate CliniCoCo firstly from a macro perspective, which demonstrates the effectiveness of the representation architecture, the introduced different types of noisy datasets, and the dynamic and iterative collaborative annotation strategy. Results in Table 2 reveal that CliniCoCo is competent for clinical coding tasks in real-world scenarios, performing robust on whether overall or chapter-level coding results. Experiments in section 4 in Supplementary Information evaluate CliniCoCo more from a human–AI collaborative perspective, demonstrating the effectiveness and figuring out the optimal technical setups of each module in the HITL framework in terms of leveraging multi-source medical knowledge. Given that Doktorchik et al.^33^ conducted a qualitative evaluation of clinically coded data quality in nine provinces across Canada, revealing incomplete and disorganised clinical documentation the main issue which limits the quality of clinical coding from health information manager perspectives, while Campbell and Giadresco.^34^ emphasised the role change of clinical coders and restructuring of coding workflow as a result of computer-assisted clinical coding technology^35^, we believe our proposed HITL framework CliniCoCo can have positive effects on transforming the current clinical coding paradigms to a great extent by effectively optimising the two main challenges in real-world scenarios: (a) how to leverage numerous incomplete and noisy EMR data without adequate annotation workload and (b) how to deeply involve clinical coders’ expertise and feedback into ACC workflow.

Furthermore, our study simulates various clinical settings in real-world scenarios and conducts quantitative analysis of the utility of CliniCoCo, aiming to figure out the trade-off between annotation efforts and their overall effectiveness in facilitating ACC. The results among simulation cases reveal the dominance of limited manual annotation workload, which, however can be significantly optimised by leveraging the automatically constructed raw and noisy-labelled datasets based on our designed multi-step contrastive learning-based strategy. Therefore, the optimal setup of annotation efforts can be quantified based on the noise ratio of EMR datasets, e.g., 1:3 manual versus automatic annotation for 30% noise HPH-50 dataset. Moreover, we highlight the effect of the quality of EMR inputs, which is directly influenced by clinicians’ note dictating skills and quality. By quantifying the noise ratio in the structured short text, we identify a cost-effective benchmark for balancing coding efficiency and quality, which is a noise ratio ranging from 0.2 to 0.3. With the objective to realise human–AI collaboration for clinical coding, apart from introducing HITL learning framework into ACC system, strengthening the training and guidelines of clinicians is another fundamental approach which should be put emphasis on^36–38^. As is revealed by the audit of clinical coding accuracy conducted by Nouraei et al.^39^ on 30,127 patients in the UK, clinical coding is prone to subjectivity, variability, and error, which in part explains the fact that data modelling has been of limited utility in predicting clinical coding. Therefore, this benchmark which includes quantitative metrics of manual annotation efforts and raw EMR quality can provide valuable insights for future improvement of EMR management and the development of policy protocols in Chinese hospitals.

In addition, in-depth clinical observations of the deployment of CliniCoCo are also conducted from a fine-grained perspective as shown in section 5 in Supplementary Information. First, CliniCoCo performs better on primary diagnosis compared to secondary diagnoses, primarily due to higher correlation and frequency in clinical text. Second, the section of medical history is observed a biggest impact on coding inference, where the subsections of present and past illness contribute the most. Third, benefiting from the flexible architecture and training strategy, CliniCoCo shows good adaptability when dealing with heterogeneous EMR contexts governed by different regulations, which suggests a potential inter-regional utility for coding among provinces in China under the transfer learning setting. Furthermore, code distributions might be different between regions and countries due to multiple factors including population composition, climate, economy, education and culture. Therefore, based on the adaptability of CliniCoCo, a comprehensive generalisability analysis of pretrained ACC models could be conducted in a diverse set of new settings, preferably across nations.

Last but not least, pilot experiments involving nine clinical coders were conducted to further verify the utility of human–AI collaborations in real-world coding scenarios. Compared with manual coding mode and “black-box” ACC mode, our HITL ACC mode can optimise both the effectiveness and efficiency of coding by clinical coders. More specifically, from inter-variability, we observed a significant optimisation for the missing mistakes in clinical text, and the collaborative manner overall helped clinical coders less susceptible to various types of noise inherent in the structured text of EMRs. From intra-variability, the most significant improvements were observed on intern coders. However, compared to senior and junior coders who excelled on collaborative manner mode, the intern in HITL mode performed slightly worse than the AI-based coding system. This may arise from the misalignment between the coding proficiency of intern coders and the performance of the AI system.

Despite extensive experiments and analyses we conducted which demonstrate the effectiveness and clinical utility of our proposed HITL framework CliniCoCo, further work can be extended for the research on human–AI collaborative clinical coding. Specifically, a more fine-grained and comprehensive case study with more clinical coders participate should be conducted to deeply analyse (a) how AI-based system impact on inter-variability and intra-variability of clinical coders, (b) how clinical coders react to the support of AI-based system in the decision-making process, and (c) what is the best collaborative setting for the clinical coding task. Additionally, the EMR heterogeneity is inherent in the Chinese health system. Different provincial regions in China follow different guidelines, writing styles, and taxonomy version. This poses a significant challenge for an ACC system to be applicable across regions, warranting much-needed future studies on enhancing and optimising the robustness and generalisation of (HITL-) ACC. In future work, we aim to further observe and take into account of the differences of coding preference, quality, and patient characteristics across regions, which may particularly benefit dedicated EMR managements for billing purpose, e.g., inter-provincial payment. As a consistent research objective of this work, based on our developed CliniCoCo system, we plan to further invite clinical coders in Chinese tertiary hospitals to conduct a case study in real-world scenarios to address the questions above.

## Methods

In this section, we illustrate the detailed architectures of the HITL collaborative strategies designed in the three key stages of CliniCoCo and the quality control simulator proposed for further quantitative analysis.

### Dynamic and Iterative collaborative annotation

Given the inferior quality of raw EMRs and the labour-intensive nature of labelling procedure, the capacity of expert coders to annotate ground truths is constrained in a small scale. Therefore, in the data preprocessing stage, a HITL collaborative annotation strategy is designed to dynamically and iteratively collaborate the manual annotation by clinical coders. With the objective to improve the labelling efficiency, the semi-automatic strategy comprises two branches. First, large-scale noisy dataset is automatically annotated based on structured short text with the adoption of the unsupervised SimCSE-based^40^ similarity computing module, aiming to perform fast and coarse annotation on wide range of raw EMRs. Second, small-scale clean dataset is manually annotated by clinical coders with the adoption of dynamic and iterative annotation strategy which adaptively allocate candidate EMRs from noisy dataset, aiming to provide dynamic knowledge orientation for local manual annotation.

#### Automatic large-scale noisy data annotation

Semi-structured raw EMRs contain pieces of structured short text, e.g., primary and secondary disease names. Such information is incomplete and flawed, which, however, can be fast-mapped to part of the coding results of the EMRs. Based on this assumption, here we construct a similarity computing module, where [CLS] token from pretrained BERT is firstly utilised to initialise the embedding of each piece of disease name text, and unsupervised SimCSE is then adopted for training of feature extractor. The loss function of the training process is shown below.

1 $${\ell }_{i}=-\log \frac{{e}^{{\rm{sim}}\left({{\bf{h}}}_{i}^{{z}_{i}},{{\bf{h}}}_{i}^{{z}_{i}^{{\prime} }}\right)/\tau }}{\mathop{\sum }\nolimits_{j = 1}^{N}{e}^{{\rm{sim}}\left({{\bf{h}}}_{i}^{{z}_{i}},{{\bf{h}}}_{j}^{{z}_{j}^{{\prime} }}\right)/\tau }}$$

where ${{h}_{i}}^{z^{{\prime} }_{i}}$ is the positive sample representation for ${{h}_{i}}^{{z}_{i}}$, which is encoded with different dropout masks. *τ* is a temperature hyperparameter and *sim*(*h*_1_, *h*_2_) is the cosine similarity.

We then utilise standard disease names from ICD taxonomy as the prototype text for each diagnosis code, and then conduct similarity computing for automatic ICD code assignment. Since the contexts in each piece of disease name is quite short and each piece may correlate with only one code, the automatic coding model can be simply trained based on unsupervised SimCSE with high accuracy. The coding results can cover a wide range of raw EMRs and thereby being used as the noisy labels of the unstructured free text in EMRs.

#### Adaptive small-scale clean data annotation

On the basis of numerous noisy-labelled EMRs, small-scale manual annotation is conducted to construct clean dataset. Here we design the HITL collaborative strategy to minimise the workload and maintain all codes distributed evenly in the clean dataset. The strategy put coders in the annotation loop and adaptively adjust the coding orientation to instruct coders deal with demanded candidate EMRs samples. Specifically, in the clean data annotation procedure, each coder is initially allocated with a patch of candidate EMRs where every ICD code appears in at least two pieces of EMR samples based on the noisy labels annotated previously. To ensure the robustness of allocated EMR samples, adjustable threshold is set for random selection from the noisy-labelled dataset based on the prediction confidence of the similarity module. Then, more patches will be iteratively selected for manual annotation. Based on the updated statistics of label distribution in the clean-labelled dataset, dynamic annotation orientation will be provided to coders, which means that upcoming patches will continuously select candidate samples containing inadequate ICD codes from the noisy-labelled dataset and then be allocated to coders. The loop of patch selection ends when every ICD code appears in at least *s* pieces of samples in clean-labelled dataset. As a result, the collaborative strategy enables adequate and even-distributed clean-labelled dataset with minimum labour required.

### 3-Step MLTC-oriented contrastive learning training

In the case of limited data annotation capacity, how to fully leverage numerous noisy-labelled raw EMR dataset and exploit their implicit rich medical knowledge is the kernel to improve the representation performance of ACC methods. Focusing on the MLTC setting of the ACC task, we propose a 3-step contrastive learning strategy to deeply enhance the representation method based on EMR datasets with different noise levels. The motivations of the proposed contrastive learning-based training are twofold. First, compared with traditional MLTC loss, binary cross entropy (BCE), which measures the mapping correlation between medical text and ICD labels, contrastive learning loss, however, measures the mapping correlation between medical text. Under the circumstance that labels of the noisy-labelled dataset are roughly correct, the noisy labels remain robust for effective measure of the distance between medical text, while may fail to correctly reflect the specific mapping correlation between medial text and codes. Second, contrastive learning loss measures the distance between medical text based on similarity computing, e.g., cosine similarity, which means that contrastive learning-based training can involve medical text with diverse ICD labels. Therefore, the size of training set can be remarkably augmented, compared with the primitive training set with limited number of candidate labels. Our proposed contrastive learning strategy effectively assess the relative similarity correlation between medical text with complex overlapping status of multiple labels. On the basis of different noise levels, samples in EMR dataset are divided into three groups and then utilised for model training and refinement step by step, i.e., incomplete raw samples without disease information, automatic noisy-labelled samples, and manual clean-labelled samples. The framework of the whole contrastive learning-based training process is shown in Fig. 3. In this subsection, we first describe the basic architecture of the feature extractor. Then we illustrate the detailed training designs in multiple steps.

#### Architecture of feature extractor

As is shown in Fig. 3, we construct a GNN-based feature extractor performing MLTC under inductive condition. Within the representation process, a piece of free text is first encoded by pretrained BERT in unit of document and words, respectively. Specifically, by adopting convolutional embedding mode, each token in the context is encoded through a sliding window in length 512. Then, a word-level graph is constructed for the text, where each node denotes a word in the context and the edges are connected according to co-occurrence between words within a fixed-size sliding window. After graph construction, a GGNN module^41^ is adopted for global information interaction, following with a readout module which aggregates the node vectors based on attention mechanism and generate final document-level representation. The readout functions are shown below.

2 $${{\bf{h}}}_{v}=\sigma \left({f}_{1}\left({{\bf{h}}}_{v}^{t}\right)\right)\odot \tanh \left({f}_{2}\left({{\bf{h}}}_{v}^{t}\right)\right)$$

3 $${{\bf{h}}}_{{\mathcal{G}}}=\frac{1}{| {\mathcal{V}}| }\sum _{v\in {\mathcal{V}}}{{\bf{h}}}_{v}+\mathrm{Maxpooling}\,\left({{\bf{h}}}_{1}\ldots {{\bf{h}}}_{{\mathcal{V}}}\right)$$

where **h**_*v*_ denotes a node vector. *f*_1_ and *f*_2_ are two multi-layer perceptrons for performing soft attention on nodes. Both averaging and maxpooling are conducted to aggregate weighted nodes for graph-level representation ${{\bf{h}}}_{{\mathcal{G}}}$.

#### Pretraining with incomplete raw samples

Apart from the noisy and clean-labelled datasets collaboratively annotated, more accessible samples are from incomplete raw EMRs which lacks structured disease names for automatic annotation. To leverage the medical knowledge in these samples, we first design an unsupervised contrastive learning loss to pretrain the feature extractor. Different from unsupervised SimCSE using dropout masks, here we utilise the aggregated representation of the feature extractor as the original sample, while utilise the [CLS] token vector as the corresponding positive sample. The detailed function is listed below.

4 $${{\mathcal{L}}}_{1}={{\mathcal{L}}}_{{\rm{us}}\_{\rm{con}}}=-\log \frac{{e}^{{\rm{sim}}\left({{\bf{h}}}_{i}^{{z}_{i}},{{\bf{h}}}_{i}^{{[CLS]}_{i}^{{\prime} }}\right)/\tau }}{\mathop{\sum }\nolimits_{j = 1}^{N}{e}^{{\rm{sim}}\left({{\bf{h}}}_{i}^{{z}_{i}},{{\bf{h}}}_{j}^{{[CLS]}_{j}^{{\prime} }}\right)/\tau }}$$

where ${h}_{i}^{{[CLS]}_{i}^{{\prime} }}$ denotes the positive pair based on [*CLS*] token. ${{\mathcal{L}}}_{1}$ denotes the loss function in the first step.

#### Training with large-scale noisy-labelled samples

Subsequently, we further train the pretrained feature extractor based on large-scale noisy-labelled samples. Existing supervised contrastive learning loss simply narrow distances between samples from the same class and push away samples from different classes, which, however, cannot deal with the complex overlapping status of medical text containing multiple labels. Therefore, here we propose the MLTC-oriented supervised contrastive learning loss where a dynamic coefficient based on the label similarity is designed to assess the relative correlation between samples in fine grain.

Specifically, given a minibatch in size *b* where *z*_*i*_ and *y*_*i*_ denotes the representation and label vector of one sample in the minibatch, respectively, we first conduct dot product of samples’ label vectors to calculate the label similarity *C*_*ij*_ between samples. Then, we conduct normalisation on *C*_*ij*_ as the dynamic coefficient *β*_*ij*_ to quantify the fine-grained relative correlation between samples in a minibatch. During each batch of training process, the dynamic coefficient is utilised to weigh each sample pair of contrastive learning loss and the whole minibatch loss *L*_*c*_*on* would be the summation of all sample pairs. As a result, the more correlated sample pairs will be assigned with higher dynamic coefficient and thereby contributing more explicitly in the numerators to be optimised closer, while the less correlated sample pair may only appear in the denominators of other terms when the dynamic coefficient is close to 0 and thereby being continuously optimised farther. Detailed loss functions are shown as below.

5 $${C}_{ij}={y}_{i}^{\top }\cdot {y}_{j}$$

6 $${\beta }_{ij}=\frac{{C}_{ij}}{{\sum }_{k\in g(i)}{C}_{ik}}$$

7 $${{\mathcal{L}}}_{{\rm{ml}}\_{\rm{con}}}=\sum _{i}\sum _{j\in g(i)}-{\beta }_{ij}\log \frac{{e}^{-d\left({z}_{i},{z}_{j}\right)/{\tau }^{{\prime} }}}{{\sum }_{k\in g(i)}{e}^{-d\left({z}_{i},{z}_{k}\right)/{\tau }^{{\prime} }}}$$

where $g(i)=\left.\right\{k| k\in \{1,2,\cdots \,,b\},k\,\ne\, i$ and *d*(. , .) denotes Euclidean distance. In addition, considering the applicability of our proposed loss for incomplete samples. Here we combine both supervised and unsupervised contrastive learning loss for the training with large-scale noisy-labelled dataset. The loss function in this step is shown below.

8 $${{\mathcal{L}}}_{2}=\gamma {{\mathcal{L}}}_{{\rm{ml}}\_{\rm{con}}}+\delta {{\mathcal{L}}}_{{\rm{us}}\_{\rm{con}}}$$

where the parameters *γ* and *δ* control the trade-off between losses. ${{\mathcal{L}}}_{2}$ denotes the overall loss function in the second step.

#### Refinement with small-scale clean-labelled samples

Being consistent with the previous training process, further refinement can be conducted based on small-scale clean-labelled samples. Given the capability of clean samples to accurately reflect the mapping correlation between medical samples and ICD codes, in this step, we further combine BCE loss with supervised and unsupervised contrastive learning loss to refine the feature extractor. In addition, a per-label attention mechanism is also adopted to improve model explainablity. The loss function in this step is shown below. As a result, the whole training process is incremental to leverage datasets with different noise levels by applying different joint loss functions in three steps.

9 $${{\mathcal{L}}}_{{\rm{BCE}}}=\sum _{l\in {\mathcal{C}}}-{y}_{l}\log \left({\hat{y}}_{l}\right)-\left(1-{y}_{l}\right)\log \left(1-{\hat{y}}_{l}\right)$$

10 $${{\mathcal{L}}}_{3}=\theta {{\mathcal{L}}}_{{\rm{BCE}}}+\gamma {{\mathcal{L}}}_{{\rm{ml}}\_{\rm{con}}}+\delta {{\mathcal{L}}}_{{\rm{us}}\_{\rm{con}}}$$

where ${\hat{y}}_{l}$ denotes the predicted probabilities of *y*_*l*_. ${{\mathcal{L}}}_{3}$ denotes the overall loss function in the third step. *θ*, *γ* and *δ* are the controllable hyperparameters.

### kNN-optimised inference involving priori expertise

With a series of contrastive learning loss designed for training, it is expected that the feature extractor has obtained the capability to distinguish the coding correlation between medical samples. Moreover, in view of limited number and the authority of clean-labelled datasets, it is necessary to fully refer to existing representative diagnostic cases so that the prediction results can better conform to priori clinical diagnosis logic and thereby being more explainable. Therefore, we further put clinical coders into the loop of model prediction by involving coders’ priori medical expertise during the code inference.

As is shown in Fig. 3, aiming to integrate the coders’ priori medical expertise, we first construct a datastore based on the samples in clean-labelled dataset. All samples in the datastore are collected in format of ${D}^{{\prime} }={\left\{\left({h}_{i},{y}_{i}\right)\right\}}_{i = 1}^{N}$, where *h*_*i*_ and *y*_*i*_ denote the feature extractor-based representation and the labels of one sample. In the inference stage, for each piece of medical text to be predicted, a kNN mechanism is conducted to retrieve top-k correlated representative samples from the datastore. The corresponding clean labels are then collected and weighed based on the relative similarity correlation. Finally, the combined kNN-based predicted results are integrated to optimise the feature extractor-based prediction results. The formulas of optimised model prediction are shown below.

11 $${\alpha }_{i}=\frac{{e}^{-d\left({h}_{i},f(x)\right)/\tau }}{{\sum }_{j}{e}^{-d\left({h}_{j},f(x)\right)/\tau }}$$

12 $${\hat{y}}_{{\rm{kNN}}}=\mathop{\sum }\limits_{i=1}^{k}{\alpha }_{i}{y}_{i}$$

13 $$\hat{y}=\lambda {\hat{y}}_{{\rm{kNN}}}+(1-\lambda ){\hat{y}}_{{\rm{Mo}}}$$

where *α*_*i*_ is the coefficient to assess the similarity between predicted medical text and retrieved samples. *d*(. , . ) denotes Euclidean distance. ${\hat{y}}_{{\rm{kNN}}}$ and ${\hat{y}}_{{\rm{Mo}}}$ denote the kNN-based and feature extractor-based prediction results, respectively. *λ* is the parameter that controls the trade-off between prediction results.

### Customised collaborative clinical decision-making

With frequent interactions between coding system and clinical coders involved, clinical decision-making stage is the most explicit presentation of human–AI collaboration. Therefore, the design of the interactive interface directly determines the practical deployment of the AI-based system in the real-world scenarios. Under the circumstance that existing ACC method cannot satisfy fully automated decision-making, interactive interface requires consistent optimisation to involve more coders’ feedback. Aiming to further improve the performance, explainability, and usability of the coding system, here we propose four customised collaborative functions which are integrated into the coder-centred interface of CliniCoCo to support coder’ decision-making from different perspectives.

#### Confidence-threshold adjustment

Based on the prediction confidence from the AI-based coding model, the threshold adjustment function is designed for coders’ to control the number of the presented candidate codes. A slider ranging from 0 to 1is laid out in the interface for customised adjustment. As a result, clinical coders can easily balance the Recall and Precision of the coding model for each specific coding task according to their preference.

#### Heatmap visualisation

The explainability of the coding system is crucial to clinical practice in terms of efficiency and trustworthiness. To help coders better understand the inference logic from EMR text, heatmap visualisation is designed based on confidences from the per-label attention mechanism. As a result, code-specific associated evidence can be highlighted in the interface to support coders read medical text.

#### Similar reference retrieval

As a consistent HITL function of kNN-optimised inference module designed for coding prediction, similar reference retrieval is developed provide the specific content and coding results of the top k retrieved EMR samples for clinical coders in decision-making stage. Although retrieved priori expertise has been integrated into the representation method, we believe that presenting the related inference sources in a more explicit way can contribute to the explainability of the system and help clinical coders better understand and trust the AI-based predicted coding results.

#### HITL interactive interface development

**Fig. 8:**
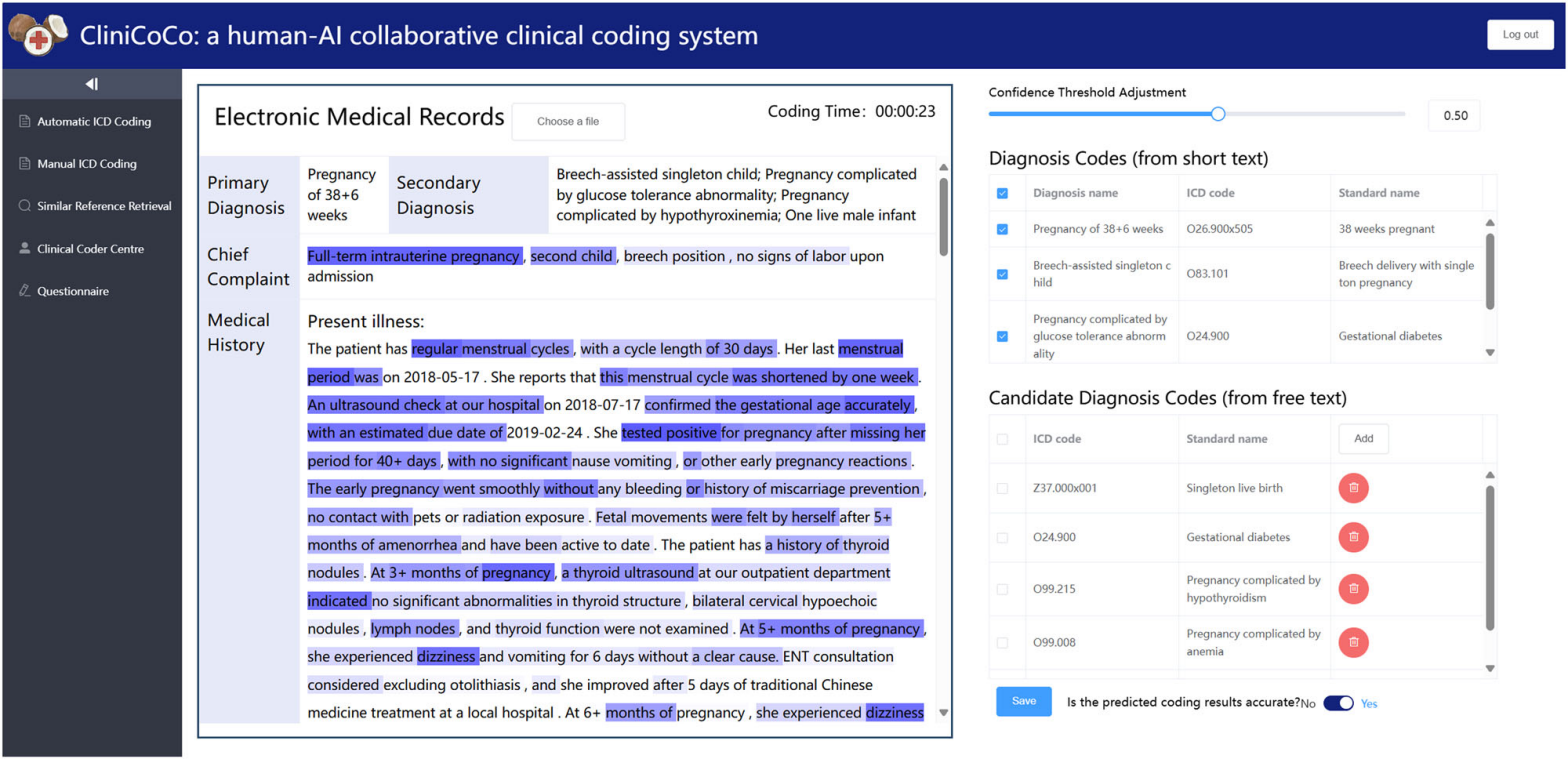
The interactive interface of CliniCoCo in the clinical decision-making stage. Our designed customised functions of threshold adjustment, heatmap visualisation, and similar reference retrieval are presented in the interface sample, respectively.

Based on our proposed HITL framework CliniCoCo, we further develop the interactive interfaces for human–AI collaborative clinical coding, which integrate all the HITL collaborative designs introduced above. Specifically, Fig. 8 presents a specific interface instance in the clinical decision-making stage where the medical text is simply translated from a real EMR sample in APH. The interfaces are built with Vue for the front-end and the python web framework Django for the web API, and are deployed for coders to conduct further case study in real-world clinical scenarios. Detailed layout of the HITL functions is confirmed according to the interview with clinical coders in Chinese tertiary hospitals. Our designed HITL modules for data annotation in the data preprocessing stage are also integrated in the system. In addition, auxiliary tools, e.g., coding timers and questionnaire, are developed for pilot experiments and further case study.

### Raw data quality control simulator for quantitative analysis

Aiming to evaluate the overall framework and the designed training strategies in diverse clinical settings, here we extendedly propose a raw data quality control simulator to quantitatively and precisely control the noise ratio and error types in the raw datasets, and then simulate the data annotation process.

Specifically, in terms of quality control, we design a rule-based automatic raw dataset construction strategy where all informally written cases of all disease names are collected from raw EMRs as a case set. Then, given the hierarchical characteristics of ICD taxonomy, we regularly generate noisy short text for each sample based on the informal case set and the gold standard labels in the datasets, which can be summarised into three types, i.e., vague, wrong, and missing compilation. First, regarding vague compilation, we randomly change the raw disease name to a informally written disease name which belongs to one of its upper-level codes. Second, regarding wrong compilation, we randomly change the raw disease name to a informally written disease name which belongs to one of its same-level codes. Third, regarding missing compilation, we simply delete the raw disease name in the EMR. The generation of noisy labels for all samples follows specific noise ratio variable.

In terms of the annotation process, with quality control in EMR datasets finished, we can then conduct the semi-automatic data annotation strategy as we designed in the data preprocessing stage, where large-scale noisy-labelled dataset and small-scale clean-labelled dataset are sequentially constructed. Specifically, here we simply use the gold standard labels to simulate manual coding operations by clinical coders.

### Experimental settings

In terms of experimental settings, in the data preprocessing stage, we collect more than 5000 mentions of disease names from the structured short text section to conduct the pretraining of the similarity computing module utilised for automatic noisy data annotation, and the accuracy of the module achieves 0.9 on the candidate codes.

In the training stage, the initial learning rate is 0.001, the number of epochs is 100, the Adam optimisation is with a 0.001 of weight decay, and the batch size is 64. Pretrained BERT-Chinese-base^42^ is utilised as the initialised encoder in the similarity computing module and the feature extractor. The size of the sliding window in the feature extractor is set as 3. The number of graph layers in the feature extractor is set as 3. Regarding the hyperparameters adjusting the proportion among loss functions and prediction outputs, *θ* is set as 1, *γ* is set as 0.5, *δ* is set as 0.02, *k* is set as 3, and *λ* is set as 0.3.

For data splits, all datasets were divided 6:2:2 for training, validation, and test set. The results in the figures and tables were derived from evaluations on test sets only. In terms of the evaluation metrics, macro and micro-averaged AUC, F1 score and Recall@k (R@k) are utilised in the experiments to measure the compared methods. Specifically, AUC denotes the area under the ROC curve (receiver operating characteristic curve), and Recall@k denotes the recall of the top-k predicted labels with the highest predictive probabilities.

### Ethics

This study was approved by the Institutional Review Board of Beijing University of Posts and Telecommunications (Approval No. BUPT-P-202401). All the clinical data was totally de-identified to ensure confidentiality and privacy.

## Supplementary information

Supplementary Information

## Data Availability

The datasets used and analysed during the current study are available from the corresponding author on reasonable request.

## Code availability

The code for dataset construction and clinical coding in this study is available from the corresponding authors upon reasonable request.
