# Supplementary material for: Optimising the paradigms of human AI collaborative clinical coding
Source: NPJ Digit Med. 2024 Dec 19;7:368. doi: 10.1038/s41746-024-01363-7 (PMC11659570; doi:10.1038/s41746-024-01363-7)
Supplement: Supplementary file 1 — Supplementary Information [file 41746_2024_1363_MOESM1_ESM.pdf]

Supplementary Information

1 Clinical Coding in Chinese Hospitals

The data utilised for clinical coding in this study is semi-structured clinical text in EMRs, which consists of structured short text describing primary and secondary diagnosis information, and unstructured long free text describing chief complaints, medical history, specialist condition, and auxiliary examination of the patient. Specifically, the medical history comprises the descriptions of present illness, past illness, personal status, family status, and menstrual status. All these structured and unstructured clinical text are written by clinicians in the summary pages of inpatient EMRs following the formatting requirements in "Basic Guidelines for Medical Record Documentation" released by NHC China[1]. Additionally, clinical coders may also refer to medical images, e.g., CTs and X-rays, and related reports when conducting clinical coding, which we omitted in this study.

Overall, as for now, hospitals in China follow ICD-10 for diagnosis coding and ICD-9-CM3 for procedure coding. However, hospitals in each provincial region follow different versions of coding standards, e.g., Beijing, Shanghai, and Jiangsu versions, which are specified by the provincial regulators, respectively. Specifically, the code number, ICD codes, and standard (Chinese) names also vary among different versions, which leads to significant heterogeneity across hospital-level EMRs. Therefore, the generalisability of clinical coding is a big challenge for ACC in real-world scenarios, which would also be a research focus in our future work.

2 Related Work on HITL Intreface Designs

Regarding research on design of interactive interfaces in the field of medical informatics, existing studies attempted to optimsie the performance, explainability, and usability of clinical decision support systems from a human-computer interaction perspective. For example, Searle et al. developed a HITL interface MedCATTrainer[2] for the biomedical NER+L task, where interfaces for active learning and configurable use-case-specific annotation are integrated, enabling users to customise the annotation requirements. Calisto et al. developed a HITL system BreastScreening-AI[3] for medical image-based breast diagnosis, which integrates an XAI method to provide radiologists with heatmaps indicating lesion severity. The literature of UI design particularly for the ACC task is, however, limited. Existing studies[4,5] mainly implemented heatmap-based approaches on clinical text based on the attention mechanism, which still lack further optimisation for more functionalities to involve clinical coders' feedback. Our framework realised a more systematic and comphrehensive three stage feedback to facilitating human and AI collaborative work.

3 Baseline Selection for Feature Extractor

Regarding baseline selection for the feature extractor in comparative experiments, CAML and LAAT were used as baselines was based on the consideration that they are the most representative deep-learning-based ACC methods. The current literature shows that LAAT performances are very robust and outstanding compared with those recently proposed ACC methods[6]. We appreciate that there are methods slightly outperform LAAT. Most of those methods benefit from utlising external third-party knowledge bases, such as UMLS, MeSH, and SNOMED, which are not directly applicable in the Chinese health system. Chen et al.'s model was chosen because it also includes a BERT-based structure, which is directly comparable to the architecture of our CliniCoCo framework. MSMN method was not chosen as a baseline because it was trained with a third-party knowledge base, UMLS, which is not directly applicable in the Chinese health system as the Chinese language contents in UMLS is limited.

4 Effectiveness of technical HITL setups in CliniCoCo

In this subsection, our analyses mainly focus on the effect of two technical HITL setups on the performance of CliniCoCo, i.e., a) the 3-step multi-label contrastive learning, and b) the kNN-based inference. Extensive ablation studies have been conducted under diverse variable settings, and the results are shown in Supplementary Table 1 and Supplementary Figure 1, respectively.

The effect of 3-step multi-label contrastive learning

First, the results shown in Supplementary Table 1 demonstrate that the supervised multi-label contrastive learning loss utilised in the second and third step do have a substantial boost of the performance improvement, which is consistent with our inference in comparative experiments. Moreover, the performance increase brought by the dynamic coefficient β is also prominent, which is close to the increase from the whole supervised contrastive learning loss, revealing that the dynamic coefficient should be a core factor which contributes to leveraging the medical knowledge implicit in multi-labels of datasets.

The effect of kNN-based inference

Based on the results shown in Supplementary Table 1, combining kNN-based inference is proven be a promising approach to optimising the feature extractor-based prediction results, with more than 1% F1 score increase in both datasets. Additionally, including contrastive learning in the kNN based component shows further improvements (+0.92% and +0.64% for HPH-50 and

Supplementary Table 1. Performance in micro-F1 score of CliniCoCo for the ablation studies regarding the effect of 3-step multi-label contrastive learning and the kNN-based inference.

	HPH-50	HPH-100
CliniCoCo	0.8420	0.8225
supervised contrastive learning loss w/o	0.7462	0.7231
dynamic coefficient β w/o	0.7638	0.7459
kNN w/o	0.8294	0.8120
kNN trained w/o contrastive learning	0.8202	0.8056

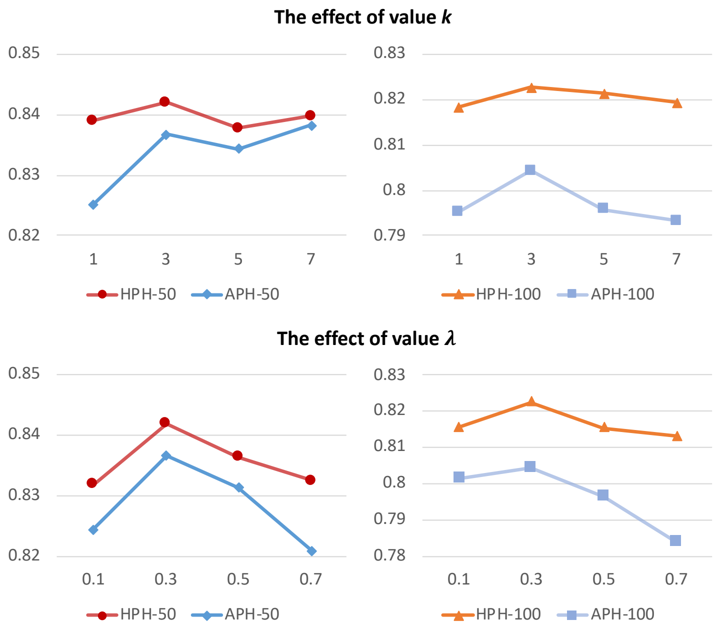

Supplementary Figure 1. Analysis on the effect of the hyperparameters on kNN-optimised inference. Results of the HPH and APH datasets with 50 and 100 labels are separately presented.

HPH-100 accordingly). Regarding the hyperparameter settings, as is shown in Supplementary Figure 1 we find that the relative optimal range for the value k is broad. With the value k set as 3, CliniCoCo performs best when the value λ which adjusts the ratio between feature-extractor-based prediction and kNN-based prediction is set as 0.3.

5 Clinical Coding Observations

In this subsection, we aim to in-depth observe the phenomena occurring in the coding results of CliniCoCo in clinical scenarios from a more fine-grained perspective.

Primary and secondary diagnosis difference

First, we specially extract the primary disease labels from the dataset and make the statistics of F1-score of CliniCoCo under the multi-class text classification s etting. As is shown in Supplementary Table 2, the diagnosis of primary disease overall outperforms the diagnosis of secondary diseases, which is reasonable considering the higher correlation between primary disease names and medical text, and the higher frequency of primary disease samples in the training set.

Impact of clinical information types on the model inference

As shown in Figure 1, an EMR contains information from various aspects including medical history, chief complaints, past illness and etc. To understand how such diverse information is utilised separately and jointly by the model in coding, we conducted attention weights based quantification analysis. Specifically, based on randomly selected EMR samples, we leverage the fine-grained weights from the word-level attention mechanism of predicted labels (visualised in Figure 8) and sum those by the section titles (i.e., information types). To avoid noises, we experimented with two thresholds of 0.4 and 0.6 on the

Supplementary Table 2. Performance in micro-F1 score of CliniCoCo for clinical observations of the prediction on primary diagnosis and the adaptability in heterogeneous EMRs, respectively.

Primary & secondary diagnosis			
	Overall	Primary	Secondary
HPH-50	0.8420	0.8704	0.8237
APH-50	0.8367	0.8623	0.8156
Heterogeneous EMR setting			
	Overall	Chapter I	Chapter E
HPH-50	0.8013	0.8172	0.8417
APH-50	0.7855	0.8106	0.7945
	Chapter G	Chapter B	Chapter F
APH-50	0.5862	0.4827	0.4574

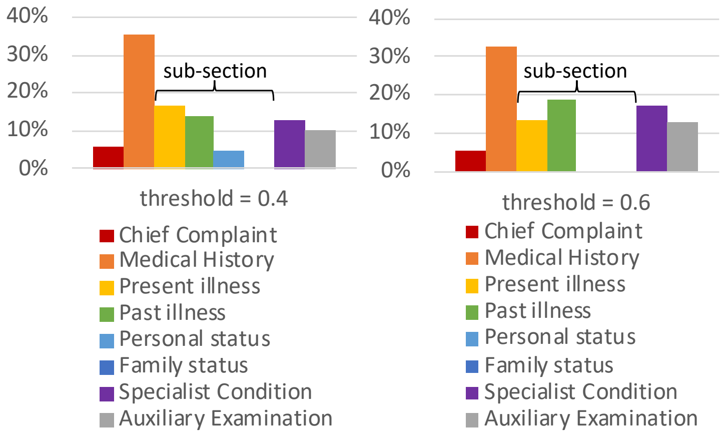

Supplementary Figure 2. The impact distribution of each section and sub-section of information in free text. Two threshold settings are selected to filter less impactful words.

normalised attention weight to conduct analysis on 'important words' only. The distributions are shown in Supplementary Figure 2, which reveal that the medical history contributes the most, while the information in the sub-sections describing present and past illness also has significant impact on coding d ecisions. Interestingly, the chief complaints, however, have shown relatively trivial impact. The observation is overall consistent between two different thresholds, except that past illness appears to play more important role when using higher threshold.

Adaptability to heterogeneity of EMR contexts

Given the multi-step characteristics of our designed contrastive learning strategy, CliniCoCo can flexibly leverage EMR datasets with different noise levels to continuously improve the representation performance. Such characteristic is convenient for different medical organisations to share and integrate medical knowledge. For example, hospital A can refine the existing feature extractor pretrained by hospital B with their own private EMR dataset, while the private EMR datasets from the two hospitals are ensured mutually inaccessible. However, the inevitable issue in real-world scenarios may occur that the management of EMRs in different medical organisations actually follow different coding guidelines, writing styles, and taxonomy version. Therefore, it is essential for us to explore the adaptability of the HITL framework to heterogeneity of EMR datasets in the format and content. The results of pilot experiments on adaptability are shown in Supplementary Table 2. Due to distinct provincial management status in current Chinese hospitals, samples in our constructed HPH and APH are heterogeneous. Therefore, we simply mutually exchange the noisy datasets utilised in the first two s teps. It can be observed that the performance of CliniCoCo decreases a little bit, but is still acceptable, which demonstrates the adaptability of our proposed HITL framework to heterogeneity of EMR datasets. This can also be reasoned to the similar code distribution between two datasets. According to the label distributions of the two datasets shown in Figure 5 , the labels in both datasets are densely distributed and overlapped in certain chapters, e.g., I, E, J, and K, etc, which is consistent with the results in Supplementary Table 2 that CliniCoCo performs

more robust on codes in the overlapped chapters whose medical correlations are better captured in the previous training steps. Furthermore, the prediction on chapter I and E prevail each other depending on their relative dominance in each pretraining dataset setting, respectively. The coding results of less overlapped chapters in the heterogeneous setting perform significantly lower than those in chapters I and E. For example, the F1 scores of the least overlapped chapters G, B, and F are overall less than 0.6.

Supplementary References

1. National Health Commission, P. R. C. Basic guidelines for medical record documentation. http://www.nhc.gov.cn/yzygj/s3585u/200904/ebe63919d67b4c65a76b3f61d1c80cd6.shtml (2010).

2. Searle, T., Kraljevic, Z., Bendayan, R., Bean, D. M. & Dobson, R. J. B. Medcattrainer: A biomedical free text annotation interface with active learning and research use case specific customisation. In *Conference on Empirical Methods in Natural Language Processing* (2019).

3. Calisto, F. M., Santiago, C., Nunes, N. J. & Nascimento, J. C. Breastscreening-ai: Evaluating medical intelligent agents for human-ai interactions. *Artif. intelligence medicine* **127**, 102285 (2022).

4. Dong, H., Suárez-Paniagua, V., Whiteley, W. & Wu, H. Explainable automated coding of clinical notes using hierarchical label-wise attention networks and label embedding initialisation. *J. biomedical informatics* **116**, 103728 (2021).

5. Teng, F. *et al.* A review on deep neural networks for icd coding. *IEEE Transactions on Knowl. Data Eng.* **35**, 4357–4375 (2023).

6. Yuan, Z., Tan, C. & Huang, S. Code synonyms do matter: Multiple synonyms matching network for automatic icd coding. In *Annual Meeting of the Association for Computational Linguistics* (2022).
